# Association of homelessness and psychiatric hospital readmission—a retrospective cohort study 2016–2020

**DOI:** 10.1186/s12888-023-04945-z

**Published:** 2023-06-23

**Authors:** Angela Russolillo, Akm Moniruzzaman, Michelle Carter, Julia Raudzus, Julian M. Somers

**Affiliations:** 1 Department of Psychiatry, St. Paul’s Hospital, Providence Health Care, 1081 Burrard Street, BC V6Z 1Y6 Vancouver, Canada; 2 Faculty of Health Sciences, Centre for Applied Research in Mental Health and Addiction, Simon Fraser University, 515 West Hastings Street, BC V6B 5K3 Vancouver, Canada

**Keywords:** Homelessness, Hospital readmission, Psychiatric disorders, Substance use disorders

## Abstract

**Background:**

A large proportion of adult psychiatric inpatients experience homelessness and are often discharged to unstable accommodation or the street. It is unclear whether homelessness impacts psychiatric hospital readmission. Our primary objective was to examine the association between homelessness and risk for 30-day and 90-day readmission following discharge from a psychiatric unit at a single urban hospital.

**Methods:**

A retrospective cohort study involving health administrative data among individuals (*n* = 3907) in Vancouver, Canada with an acute psychiatric admission between January 2016 and December 2020. Participants were followed from the date of index admission until censoring (December 30, 2020). Homelessness was measured at index admission and treated as a time-varying exposure. Adjusted Hazard Ratios (aHRs) of acute readmission (30-day and 90-day) for psychiatric and substance use disorders were estimated using multivariable Cox proportional hazards regression.

**Results:**

The cohort comprised 3907 individuals who were predominantly male (61.89%) with a severe mental illness (70.92%), substance use disorder (20.45%) and mean age of 40.66 (SD, 14.33). A total of 686 (17.56%) individuals were homeless at their index hospitalization averaging 19.13 (21.53) days in hospital. After adjusting for covariates, patients experiencing homelessness had a 2.04 (1.65, 2.51) increased rate of 30-day readmission and 1.65 (1.24, 2.19) increased rate of 90-day readmission during the observation period.

**Conclusions:**

Homelessness was significantly associated with increased 30-day and 90-day readmission rates in a large comprehensive sample of adults with mental illness and substance use disorders. Interventions to reduce homelessness are urgently needed.

**Question:**

Is homelessness associated with risk for 30-day and 90-day psychiatric hospital readmission?

**Findings:**

In this retrospective cohort study of 3907 individuals, homelessness at discharge was associated with increased 30-day and 90-day psychiatric readmission.

**Meaning:**

Housing status is an important risk factor for hospital readmission. High-quality interventions focused on housing supports have the potential to reduce psychiatric readmission.

**Supplementary Information:**

The online version contains supplementary material available at 10.1186/s12888-023-04945-z.

## Introduction

Homelessness is a widespread health and social concern across North America. In Canada more than 235,000 people experience homelessness in any given year, and thousands more reside in precarious or inadequate housing [1]. Individuals facing homelessness experience an increased burden of illness, mortality and barriers to obtaining health care [2]. A significant proportion of individuals struggling with homelessness also suffer from substance dependence and other concurrent mental illnesses [3]. Individuals with mental illness and addictions are more likely to experience repeated episodes and longer periods of homelessness [4], as well as to require more health and social services [5, 6]. Despite increased evidence of the effect of social factors on health care outcomes, many individuals continue to face poor living conditions and limited community supports creating a reliance on acute health services.

Readmission to hospital is an important indicator of health system performance, representing discontinuity in care and exacting high costs. In Canada, readmissions to hospital costs more than 2.3 billion annually with 1 in 11 patients readmitted within 30 days of discharge [7]. Several risk factors are associated with hospital readmission including age, income, illness severity, substance use, and prior hospitalization or emergency room visits [8, 9]. People with psychiatric disorders have some of the highest readmission rates among all hospitalized patients. The 30-day readmission rate for people with mental health and substance use disorders was 13.8% in 2022, compared to the general Canadian population rate of 9.3% [7]. Early readmission risk (within 30 days) among psychiatric populations is further complicated by social-structural vulnerabilities and diagnostic variables, with concurrent substance use and serious mental disorders (e.g., schizophrenia and bipolar disorder) associated with increased 30-day readmission risk [10].

Homeless individuals are significantly more likely to be hospitalized and present with psychiatric and substance use disorders than stably housed individuals [11, 12]. In Ontario, Canada, Saab et al. 2016 [13] found that homeless patients had nearly four times the odds of being readmitted within 30-days as compared to low-income controls. Once admitted to hospital this population often requires more specialized care and longer lengths of stay largely attributable to illness severity and psychosocial needs [14, 15] resulting in increased costs. For example, homeless patient admissions on the psychiatric service at a Toronto, Canada hospital cost $1058 more than housed patient admissions, even after adjusting for length of stay [16]. Moreover, individual recovery and social re-integration are challenging for the majority of individuals following discharge and are often complicated by poor access to treatment and support services resulting in substantial unmet needs [17]. As a result, homeless individuals face an increased risk for early and often frequent return to hospital becoming stuck in a costly and burdensome "revolving door" associated with significant economic and health consequences [18, 19].

Vancouver’s Downtown East Side (DTES) neighborhood is known as one of the largest open drug scenes in North America, with a high prevalence of poverty, mental illness and overdose mortality [20, 21]. Individuals residing in this region have some of the highest rates of homelessness and concurrent disorders in Canada [22] and these factors increase the risk of hospitalization and emergency department use [23]. While existing evidence suggests the most robust predictor of readmission is the number of previous hospitalizations [24]; gaps persist in the literature on other risk factors associated with readmission, especially among psychiatric populations who present with complex health and social needs. A growing body of observational research has identified homelessness as an independent risk factor for general hospital readmission [12, 25, 26]; however, [13] understanding the specific impact on psychiatric acute services is increasingly critical. Prior studies have found that between 7.2% to 15% of patients hospitalized for a mental health reason had an early readmission [27–29], but only a small number of published studies have assessed the influence of housing status on readmission following an index psychiatric hospitalization. These studies have shown a relationship between homelessness and readmission following acute psychiatric hospitalization; however, there is wide variation in the magnitudes of effect for readmissions [30, 31]; and little is known about the relationship between homelessness and psychiatric discharge particularly in settings known to have patients with high rates of psychiatric comorbidities and medical needs such as Vancouver’s DTES. A large Canadian-multi site study, examined 30-day hospital readmission [32]; however only 2.3% of their population was homeless, and it’s not clear that a similar relationship would be observed in other settings or what the relationship is between homeless and longer periods of follow-up. Moreover, existing research involves small samples sizes [33], limited diagnostic subgroups [32, 34]and it remains unclear how generalizable these findings are to broader psychiatric populations or geographic regions [32].

Our primary objective was to examine the association between homelessness and risk for 30-day and 90-day readmission to hospital following psychiatric discharge. We aimed to provide clinical and sociodemographic details of the population and hypothesized that in adjusted models homeless individuals would have higher rates of short-term (30-day and 90-day) readmission following acute psychiatric hospitalization.

## Methods

### Design, participants and data source

This retrospective cohort study used health administrative data for adults with a psychiatric admission to an urban hospital in Vancouver, British Columbia during a 4-year period between January 1, 2016 and December 30, 2020. The patient’s first discharge during that time period determined the index admission. Our study site provides inpatient and emergency services to the catchment area that includes the DTES. Patients were excluded from the study if they had an invalid personal health number, invalid or missing age or sex data, were younger than 17 years of age, died during the psychiatric hospitalization or transferred to another hospital at the time of discharge. Sociodemographic variables (age, sex, and ethnicity) were obtained from electronic medical records. Hospitalization data were obtained from the Ministry of Health’s Discharge Abstract Database, which includes information related to each acute hospital separation. The study exclusively used retrospective deidentified administrative records and was reviewed and approved by the University of British Columbia – Providence Health Care (UBC-PHC) Research Ethics Board. This study followed the Strengthening the Reporting of Observational Studies in Epidemiology (STROBE) reporting guideline.

### Measures

Data on the main exposure (homelessness) were extracted from electronic medical records during the index hospitalization. Homelessness was coded at each admission as part of routine assessment (no formal instrument or tool was used) and was defined as having no fixed address. Homelessness was treated as a time-varying exposure (i.e., homeless status was not constant throughout follow-up). The main outcome was 1 or more readmissions to a single urban hospital within 30-days or 90-days following acute psychiatric hospitalization. The 30-day outcome is considered a benchmark indicator for assessing coordination and continuity of care following hospital discharge and is used by several health and government agencies (e.g., Canadian Institute for Health Information). All hospitalization records included diagnostic codes representing the most responsible diagnoses for each participant’s acute psychiatric hospitalization. The present study used *International Statistical Classification of Diseases and Health Problems, Tenth Revision, Canada* (*ICD-10-CA*) to determine the most responsible diagnosis, a modified version of *ICD-10*, developed by Canadian Institute of Health Information and used across Canada. The following ICD-10-CA codes were used to determine the most responsible diagnoses for hospitalizations associated with: substance use disorders (F10-F19); Schizophrenia (F20-F29); and Bipolar Disorder (F30-F39).

### Statistical analysis

We used descriptive statistics (counts and proportions for nominal variables) and measures of central tendency (mean and standard deviation (SD) or median and interquartile range (IQR) for continuous variables to characterize the sample. Time at risk started when participants were discharged at their index visit (1^st^ recorded hospital visit) during the observation period and ended at the 1^st^ anniversary since their index release or December 30, 2020 (whichever occurred first). Time spent in hospital was excluded from time at risk. We chose time-to-event analysis because our outcome of interest was not only the occurrence of an event (readmission), but also when it occurred. Due to the recurrent nature of the outcome variable, we used the Anderson-Gill (AG) counting process method [35], an extension of the Cox model [36], to estimate the hazard of hospital readmission associated with homelessness.

During the model building process, we assessed the proportional hazards assumption of homelessness and other covariates using Schoenfeld residuals [37]. We found a violation of the assumption of proportionality for homelessness in the univariate Cox model and refitted the univariate Cox model with the interaction terms between homelessness and follow-up time at three non-overlapping temporal intervals. This model provided estimates of hazard ratios (HR) for readmission in each temporal interval (≤ 30 days, 31 to ≤ 90 days & 91 to ≤ 365 days).

The multivariable Cox proportional hazards regression model included homelessness and temporal interaction terms as well as controlling variables selected based on their established associations with readmission: age at index visit (continuous measure), gender (men, women and unknown/other); length of stay at index visit (continuous measure), substance use disorder (no vs. yes) as reason of stay at index visit and severe mental illness (either schizophrenia or bipolar disorder) as reason of stay at index visit (no vs. yes). Existing literature highlights the association between age, length of stay, male gender, substance use comorbidities and severe mental illness as independently associated with increased psychiatric readmission risk [38–42].

As an effect size, we report the adjusted Hazard Ratio (aHR) and 95% confidence interval (CI). We chose the conventional α level (2-tailed *P* < 0.05) to interpret the significance of estimated parameters. To account for dependencies between events within the same individual, we used the robust variance estimator to estimate standard errors for the parameters [43]. We conducted sensitivity analyses to examine our primary outcome as a fixed covariate. Additional details of the analytic methods are presented in previous research [44].

## Results

The cohort was comprised of 3907 individuals who were predominantly male (61.89%) with a severe mental illness (70.92%), substance use disorder (20.45%) and mean age of 40.66 years (SD: 14.33). A total of 686 (17.56%) individuals were homeless at their index hospitalization averaging 19.13 (SD: 21.53) days in hospital. Baseline sociodemographic and readmission details for the eligible sample are presented in Table 1.

**Table 1:** Socio-demographic and clinical characteristics of included patients (*n* = 3,907)

Socio-demographic characteristics	Mean (SD) / n (%)
Age at index visit	
Mean (SD)	40.66 (14.33)
Median (IQR)	39.0 (29.00, 51.00)
Min, Max	17.00, 96.00
Gender, n (%)	
Women	1,474 (37.73)
Men	2,418 (61.89)
Other/unknown	15 (0.38)
Length of stay at index visit	
Mean (SD)	19.13 (21.53)
Median (IQR)	12.00 (5.00, 26.00)
Min, Max	1.00, 195.00
**Number of readmissions during the 1st year of follow-up period**	
Mean (SD)	0.38 (0.53)
Min, Max	0,7
Total	1,494
**Readmissions during the 1st year of follow-up period, n (%)**	
1	955 (24.44)
2	317 (8.11)
3	131 (3.35)
4	59 (1.51)
5	23 (0.59)
6 +	7 (0.18)
Length of stay at 1^st^ readmission (n = 955) during the 1st year of follow-up period	
Mean (SD)	18.80 (22.50)
Median (IQR)	12.00 (4.00, 26.00)
Min, Max	1.00, 181.00
Homelessness status at index visit, n (%)	
No	3,221 (82.44)
Yes	686 (17.56)
Most responsible diagnosis for hospital stay at index visit	
**Schizophrenia, *n (%)***	
No	2,028 (51.91)
Yes	1,879 (48.09)
**Bipolar, *n (%)***	
No	2,987 (76.5)
Yes	920 (23.55)
**Schizophrenia or bipolar, *n (%)***	
No	1,108 (28.36)
Yes	2,799 (71.64)
**Substance use disorder*, n (%)***	
No	3,111 (79.63)
Yes	796 (20.37)

*IQR* Inter-quartile Range, *Min* Minimum, *Max* Maximum, *SD* Standard Deviation

After adjusting for covariates, the aHR for readmission among patients experiencing homelessness was 2.04 (IQR: 1.65, 2.51) within 30-days and 1.65 (IQR: 1.24, 2.19) within 90-days following discharge. Moreover, risk for readmission remained significant between 91 to 365 days following the index hospitalization (Table 2).

**Table 2:** Extended Cox regression analysis estimating the hazard associated with homelessness^a^ for readmission to a large urban hospital in Vancouver, British Columbia, 2016–2020 (*n* = 3,907)

**Follow-up time**	**Homeless status**	**Total events**	**Total PDs**	**Incidence per PY**	**Unadjusted HR**^**b**^ **(95% CI**^**c**^**)**	**Adjusted HR**^**d**^ **(95% CI)**
≤ 30 days	No Yes	340 149	91,713 19,500	1.35 2.79	Reference 2.05 (1.67, 2.53)	Reference 2.04 (1.65, 2.51)
31–90 days	No Yes	237 85	181,213 39,188	0.48 0.79	Reference 1.66 (1.25, 2.21)	Reference 1.65 (1.24, 2.19)
91–365 days	No Yes	504 179	756,492 170,294	0.24 0.38	Reference 1.58 (1.28, 1.95)	Reference 1.56 (1.26, 1.94)
Overall	No Yes	1,081 413	1,029,418 228,982	0.38 0.66		

*CI* Confidence Interval, *HR* Hazard Ratio, *PDs* Person-Days, *PY* Person-Year

^a^Homelessness was used as time-varying covariate

^b^This cox model includes homelessness and the interaction terms with time (at 30 & 90 days)

^c^95% CIs and both unadjusted and adjusted hazard ratios were estimated using Robust Standard Errors

^d^The multivariable Cox model was controlled for age at index visit (continuous measure), gender (men, women & unknown/other); length of stay at index visit (continuous measure), substance use disorder (no vs. yes) as reason of stay at index visit and severe mental illness (either schizophrenia or bipolar disorder) (no vs. yes) as reason of stay at index visit

The association between readmission and homelessness were further investigated in sensitivity analyses (see Supplementary Material).

## Discussion

Homelessness at discharge was associated with a statistically significant and clinically meaningful increase in readmission rates in a large comprehensive sample of adults diagnosed with mental illnesses and substance use disorders. Our findings confirmed our hypothesis and extended knowledge by demonstrating the risk for readmission within 30-days and 90-days following discharge from acute psychiatric hospitalization.

Our results confirm a very high level of homelessness among patients discharged from an urban teaching hospital serving the DTES neighbourhood of Vancouver, British Columbia. While 17.5% of our sample was deemed homeless, only 2.3% of participants in a comparable study were identified as homeless [32], representing an 8.5-fold difference in homelessness status at discharge. Despite the difference in prevalence of homelessness in our samples we had similar rates for 30-day readmission 1.43 aHR and 2.04 aHR, respectively, which adds to confidence in our results and enhances generalizability of our findings. Our results are important, as early readmission is considered a negative outcome from a health system perspective. Several clinicians and researchers have developed interventions aimed at reducing early readmission, yet we continue to see increasing risk for readmission following discharge from acute psychiatric hospitalization. The transition from an acute psychiatric inpatient stay to community living represents a critical period in which patients frequently experience competing priorities such as obtaining food, clothing, and housing [45]. During this immediate post-discharge period unplanned readmissions are common. Findings from a large retrospective cohort in the United States (US), found that homeless adults were 54% more likely to be readmitted to a psychiatric unit within 30-days of discharge [46] when compared to housed adults. While rates of readmission to hospital are frequently used as performance indicators and considered markers of health care quality (e.g., incomplete treatment and inadequate coordination of health services following discharge)[47] they may also reflect deficiencies in social and community services. Results from a recent prospective cohort study reported that few participants indicated their readmission was due to unattainable health care after discharge but rather the result of barriers accessing general assistance and services for basic social determinants of health (e.g., housing, employment, etc.) [48]. Our results reinforce these previously reported findings and indicate that homelessness is associated with both an immediate (30-day and 90-day) and prolonged (91 to 365 days) risk for readmission. Collectively, the growing body of research that our study contributes to indicates a need to strengthen nonmedical support systems in order to reduce preventable readmissions among homeless individuals discharged from acute psychiatric care.

Research in the US and Canada has shown that providing specific forms of housing and support to homeless individuals is associated with fewer hospital days and ED visits [49–51]. Specifically, supported scattered site housing has demonstrated robust results in Canadian randomized controlled trials including large reductions in emergency department visits [52], diversion from crime [53] and improved quality of life [54] among homeless adults diagnosed with psychiatric and substance use disorders.  While current practices prioritize timely post discharge care, high-quality research has demonstrated that timely follow-up has no significant effect on reducing the risk of readmission among those who experience substance use, mental illness, and who remain homeless [55]. Our findings support the growing evidence base highlighting the importance of housing as a modifiable and protective factor against readmission.

Housing is considered a crucial social determinant of health and being homeless or unstably housed not only impacts the individual experiencing inequities, but also has substantial economic impacts. Latimer and colleagues [56] conducted an economic analysis of public services received by people experiencing homelessness, addiction, and mental illness in Canada, concluding that costs ranged between $53,000 to $58,000 per person/per year in Canada’s three largest cities (Vancouver, Toronto and Montreal). Additionally, in Vancouver, 12–30% of annual per person costs were directly related to psychiatric hospitalizations while less than 5% of annual per-person costs went to supportive housing. Canadian research has demonstrated that scattered-site recovery-oriented housing offsets between 69% [57] and 96% [58] of the cost of intervening, a rate of return that is uncommon in other domains of health and social spending. The longstanding narrative that more health care is needed for patients experiencing homelessness and psychiatric illness undermines the importance of other key social determinants of health. Even after adjustment for other risk factors including length of stay and severe mental illness, homelessness was associated with increased readmission in our sample. Policy addressing permanent supportive housing is critical to the health of individuals and the long-term stability of health system resources. Our findings demonstrating that homelessness is related to readmission have high importance to hospital administrators and policy makers particularly in settings that have a high percentage of individuals who are homeless with psychiatric and substance use comorbidities. Evidence from high-quality randomized controlled trials is available to guide policies and practices [59, 60].

Our study has several strengths, including its large sample and use of comprehensive hospitalization administrative data; however, we also have limitations to consider. First, the use of a Canadian population with health services that are publicly funded may limit the generalizability of our results to other settings and jurisdictions. Second, our reliance on administrative data is subject to bias related to missing or incomplete records, including omissions of individuals who are provisionally accommodated and those who are at risk of homelessness. Third, some key data such as race/ethnicity, chronic health conditions, and ancillary services, which may have shown associations with early readmission were not available in our dataset. Lastly, our outcome was restricted to admissions recorded at a single hospital, and therefore it failed to account for hospital admissions outside of the catchment area in Vancouver, British Columbia.

## Conclusion

Our results demonstrate an association between homelessness and psychiatric hospital readmissions. Specific interventions to reduce the burden of readmissions among individuals experiencing homelessness, substance use and mental illness have been empirically supported by randomized trials and are urgently needed. Given the increased risk for acute service use among individuals with mental illness and substance use following discharge, our findings reinforce the urgent need to revise policies and practices addressing housing and social care. A body of high-quality evidence demonstrates the effectiveness, feasibility, and cost-effectiveness of practices that improve health outcomes and support recovery among people experiencing complex psychiatric and substance use issues and homelessness.

## Supplementary Information

**Additional file 1.** 

## Data Availability

The datasets analysed during the current study are not publicly available due privacy reasons but are available from the corresponding author on reasonable request.
