# Supplementary material for: Association of homelessness and psychiatric hospital readmission—a retrospective cohort study 2016–2020
Source: BMC Psychiatry. 2023 Jun 23;23:459. doi: 10.1186/s12888-023-04945-z (PMC10288711; doi:10.1186/s12888-023-04945-z)
Supplement: Supplementary file 1 — Additional file 1. [file 12888_2023_4945_MOESM1_ESM.docx]

**Supplementary Material**

Angela Russolillo, PhD; Akm Moniruzzaman, PhD; Michelle Carter, RN, MSN; Julia Raudzus, MD; Julian M Somers, PhD. Association of Homelessness and Psychiatric Readmission - A Retrospective Cohort Study 2016-2020

**eTable 1.** Extended Cox Regression analysis estimating the hazard associated with homelessness for readmission to an urban hospital, 2016-2020 (n=3,907)

**eTable 2.** Full Model-Extended Cox Regression analysis estimating the hazard associated with homelessness readmission to an urban hospital, 2016-2020 (n=3,907)

**eTable 3**. Most Responsible Diagnoses Codes for patients at index admission (n=3,907)

**eTable 4**. Most Responsible Diagnoses Codes for patients with a readmission(s) (n=1,494)

This supplementary material has been provided by the authors to give readers additional information about our work.

**eTable 1: Extended Cox regression analysis estimating the hazard associated with homelessness^[[1]](#footnote-1)^ for re-hospitalizations in a tertiary urban hospital, 2016-2020 (n=3,907)**

| **Follow-up time** | **Homeless status** | **Total events** | **Total PDs** | **Incidence per PY** | **Unadjusted HR^^[[2]](#footnote-2)^^**  **(95% CI**^^[[3]](#footnote-3)^^**)** | **Adjusted HR^^[[4]](#footnote-4)^^**  **(95% CI)** |
| --- | --- | --- | --- | --- | --- | --- |
| ≤30 days | No  Yes | 357  132 | 92,094  19,119 | 1.41  2.52 | Reference  **1.78 (1.43, 2.20)** | Reference  **1.75 (1.41, 2.18)** |
| 31-90 days | No  Yes | 255  67 | 181,828  38,573 | 0.51  0.63 | Reference  1.24 (0.91, 1.69) | Reference  1.22 (0.89, 1.67) |
| 91-365 days | No  Yes | 525  158 | 758,867  167,919 | 0.25  0.34 | Reference  **1.36 (1.09, 1.70)** | Reference  **1.34 (1.07, 1.68)** |
| Overall | No  Yes | 1,137  357 | 1,032,789  225,611 | 0.40  0.58 |  |  |

CI: Confidence Interval; HR: Hazard Ratio; PDs: Person-Days; PYs: Person-Years

**eTable 2: Full Model – Extended Cox regression analysis estimating the hazard associated with homelessness^[[5]](#footnote-5)^ for re-admission to an urban hospital, 2016-2020 (n=3,907)**

| **Independent variables** | **Unadjusted HR^^[[6]](#footnote-6)^^**  **(95% CI^^[[7]](#footnote-7)^^)** | **Adjusted HR^^[[8]](#footnote-8)^^**  **(95% CI)** |
| --- | --- | --- |
| **Homelessness^[[9]](#footnote-9)^ * ≤30 days** | **2.05 (1.67, 2.53)** | **2.04 (1.65, 2.51)** |
| **Homelessness * 31-90 days** | **1.66 (1.25, 2.21)** | **1.65 (1.24, 2.19)** |
| **Homelessness * 91-365 days** | **1.58 (1.28, 1.95)** | **1.56 (1.26, 1.94)** |
| **Age at index visit (per year)** | **0.99 (0.99, 1.00)** | **0.99 (0.99, 1.00)** |
| **Gender**  Women  Men  Other/unknown | Reference  **1.25 (1.07, 1.45)**  1.57 (0.78, 3.16) | Reference  1.16 (0.99, 1.35)  1.61 (0.81, 3.19) |
| **Length of stay at index visit (per day)** | 1.00 (0.99, 1.00) | 1.00 (0.99, 1.00) |
| **Substance use Disorder status at Index visit**  No  Yes | Reference  **0.85 (0.73, 0.99)** | Reference  1.13 (0.83, 1.54) |
| **Severe (either schizophrenia or bipolar disorder) mental illness status at Index visit**  No  Yes | Reference  **1.34 (1.16, 1.54)** | Reference  **1.63 (1.23, 2.17)** |

**eTable 3: Most Responsible Diagnoses Codes for patients at index admission (n=3,907)**

| **Most Responsible Diagnosis (MRD)** | **Frequency** | **Percent** |
| --- | --- | --- |
| F29: Unspecified nonorganic psychosis | 616 | 15.77 |
| F209: Schizophrenia, unspecified | 605 | 15.49 |
| F155: Mental and behavioural disorders due to use of other stimulants including caffeine, psychotic disorder | 400 | 10.24 |
| F312: Bipolar affective disorder, current episode manic with psychotic symptoms | 280 | 7.17 |
| F252: Schizoaffective disorder, mixed type | 273 | 6.99 |
| F322: Severe depressive episode without psychotic symptoms | 140 | 3.58 |
| F259: Schizoaffective disorder, unspecified | 123 | 3.15 |
| F603: Emotionally unstable personality disorder | 104 | 2.66 |
| F311: Bipolar affective disorder, current episode manic without psychotic symptoms | 99 | 2.53 |
| F200: Paranoid schizophrenia | 96 | 2.46 |
| F329: Depressive episode, unspecified | 92 | 2.35 |
| F319: Bipolar affective disorder, unspecified | 77 | 1.97 |
| F195: Mental and behavioural disorders due to multiple drug use and use of psychoactive substances, psychotic disorder | 66 | 1.69 |
| F102: Mental and behavioural disorders due to use of alcohol, dependence syndrome | 46 | 1.18 |
| F332: Recurrent depressive disorder, current episode severe without psychotic symptoms | 44 | 1.13 |
| Other | 846 | 21.7 |
| **Total** | **3907** |  |

**eTable 4**. **Most Responsible Diagnoses Codes for patients with readmission(s) (n=1,494)**

| **Most Responsible Diagnosis (MRD)** | **Frequency** | **Percent** |
| --- | --- | --- |
| F209: Schizophrenia, unspecified | 296 | 19.81 |
| F252: Schizoaffective disorder, mixed type | 195 | 13.05 |
| F155: Mental and behavioural disorders due to use of other stimulants including caffeine, psychotic disorder | 179 | 11.98 |
| F29: Unspecified nonorganic psychosis | 178 | 11.91 |
| F312: Bipolar affective disorder, current episode manic with psychotic symptoms | 93 | 6.22 |
| F259: Schizoaffective disorder, unspecified | 83 | 5.56 |
| F200: Paranoid schizophrenia | 68 | 4.55 |
| F603: Emotionally unstable personality disorder | 39 | 2.61 |
| F319: Bipolar affective disorder, unspecified | 36 | 2.41 |
| F195: Mental and behavioural disorders due to multiple drug use and use of psychoactive substances, psychotic disorder | 23 | 1.54 |
| F311: Bipolar affective disorder, current episode manic without psychotic symptoms | 21 | 1.41 |
| F205: Residual schizophrenia | 18 | 1.2 |
| F322: Severe depressive episode without psychotic symptoms | 18 | 1.2 |
| F250: Schizoaffective disorder, manic type | 15 | 1 |
| Other | 232 | 15.5% |
| Total | 1494 |  |

1. -Homelessness at index visit was used as a fixed covariate. [↑](#footnote-ref-1)
2. -This cox model includes homelessness and the interaction terms with time (at 1 & 2 years). [↑](#footnote-ref-2)
3. -95% CIs and both unadjusted and adjusted hazard ratios were estimated using Robust Standard Errors. [↑](#footnote-ref-3)
4. - The multivariable Cox model was controlled for age at index visit (continuous measure), gender (men, women & unknown/other); length of stay at index visit (continuous measure), substance use disorder (no vs. yes) as reason of stay at index visit and severe (either schizophrenia or bipolar disorder) mental illness (no vs. yes) as reason of stay at index visit. [↑](#footnote-ref-4)
5. -Homelessness was used as time-varying covariate. [↑](#footnote-ref-5)
6. -This cox model includes homelessness and the interaction terms with time (at 30 & 90 days). [↑](#footnote-ref-6)
7. -95% CIs and both unadjusted and adjusted hazard ratios were estimated using Robust Standard Errors. [↑](#footnote-ref-7)
8. -The multivariable Cox model was controlled for age at index visit (continuous measure), gender (men, women & unknown/other); length of stay at index visit (continuous measure), substance use disorder (no vs. yes) as reason of stay at index visit and severe mental illness (either schizophrenia or bipolar disorder) (no vs. yes) as reason of stay at index visit. [↑](#footnote-ref-8)
9. - * Indicated interaction term between follow up time and homelessness status. [↑](#footnote-ref-9)
